# Communication training is inadequate: the role of deception, non-verbal communication, and cultural proficiency

**DOI:** 10.1080/10872981.2020.1820228

**Published:** 2020-09-17

**Authors:** Aaron D. Baugh, Allison A. Vanderbilt, Reginald F. Baugh

**Affiliations:** a Pulmonary, Critical Care, Allergy, Sleep Medicine, Department of Internal Medicine, University of California San Francisco Medical School, San Francisco, CA, USA; b Department of Emergency Medicine, Fulton County Health Center, Wauseon, OH, USA; c Department of Surgery, University of Toledo College of Medicine and Life Sciences, Toledo, OH, USA

**Keywords:** Communication skills training, medical school, medical school curricula, social justice

## Abstract

In this commentary, we argue that the limited experiential exposure of medical students to different cultures makes the instruction devoted to communication skills inadequate. The relationship of these dynamics to honesty in clinical encounters is explored.

Absent significant experiential exposure to differing group cultures to counter the natural tendency to favor one’s own, discrimination prevails. Knowledge or awareness of cultural differences does not necessarily equate to communication proficiency. Critically, interactions based on lived experience offer a deeper knowledge and understanding of culturally meaningful nuances than that imparted through other formats. Medical students’ lack of experiential exposure to different cultures results in communication miscues. When the stakes are high, people detect those miscues diminishing trust in the doctor-patient relationship. Greater experiential cultural exposure will enhance the facility and use of culturally specific communication cues. At its core, the requisite transformation will require medical students to adapt to other cultures and greater representation by marginalized and stigmatized populations not only among the studentry but staff and faculty. The time is now to ensure that the physicians we produce can care for all Americans. What cannot be taught must be identified by the selection process. Competence with half the population is a failure for American medicine.

Healthcare systems of every society have an interest in improving the capacity of their physicians to deliver culturally competent care. The United Nations has recognized addressing the inequalities faced by marginalized populations as part of its Sustainable Development Goals [1]. Atop these pressures, forcibly displaced populations have increased dramatically since 2012. Populations under the mandate of the United Nations Refugee Agency expanded by over 70% during that period [2]. Of these, one study found that over 20% of such vulnerable populations had given up seeking medical care entirely due to the difficulties they encountered, attesting to the importance of culturally sensitive care [3]. From 1980 to 2000, diversity increased on average across even the relatively homogenous countries of the OECD [4].

In spite of this evident need, our provision of care leaves much to be desired. Marginalized groups are more likely to experience culturally insensitive health care and dissatisfaction with their healthcare: healthcare experiences that have been linked to poorer health outcomes [5]. Studies of vulnerable populations from Australia [6], Uganda [7], to the U.S [8,9]. have identified racism and culturally insensitive care as a major cause of dissatisfaction with the care they receive and under-utilization. This commonality is in part due to cross-cultural communication deficiencies. In this commentary, we argue that the failure of communication that underlies these issues is rooted directly in changing demographics and the attitudes of physicians. We argue that optimal cross-cultural communication comes from within where knowledge of cultural differences and modes of communication are present with the desire to communicate effectively.

Socialization during childhood imparts values and social tendencies [10] that guide behavior later in life, despite one’s education, later experiences, and changing life circumstances [11]. The resilience of biographical and cultural teachings represents a special challenge to physicians. To function effectively they must routinely interact with patients from different backgrounds and cultures. The challenges are similar regardless if physicians are caring for indigenous, religious minorities, LGBTQ+, physically disabled, immigrant, or socioeconomically disadvantaged populations. Like most countries, in the U.S., 75%-80% of the medical students from the top two quintiles of parental income in America [12]. Students often lack cross-cultural exposure due to self-imposed residential, economic, and cultural segregation [13–15]. In the absence of significant experiential exposure [16,17] to different cultures, the tendency to favor one’s own predominates [17–19]. The failure to have had meaningful intercultural exposures presages a negative effect on intercultural attitudes with diversity exposure [20]. Accordingly, about 20% of medical students have worse implicit biases against marginalized populations after medical school than before despite our educational efforts [21]. The challenges in inspiring such candidates to adopt the professional standard of compassionate regard for all patients are evident. Growing wealth inequality will only make the situation worse. Absent a socially verified shared reality, the interpretation of cultural information correctly is unlikely to provide a basis for effective communication [22].

Communication, a fundamental pillar of human social interaction and the practice of medicine, requires speaker-listener alignment. Effective communication between two people requires the overlap of verbal and non-verbal cues which might be either culturally specific or general. Healthcare should aspire to something higher still: the creation of a shared reality between the physician and patient. Absent a socially verified shared reality, the interpretation of cultural information correctly is unlikely to provide a basis for effective communication [23]. In this model, physician-patient dyads adopt a common view of a subject that both facilitates action and builds trust between them [23]. Cultural influences arising from not only nationality, migration status, race, ethnicity, language, or religion, but also age, gender identity, sexual orientation, socioeconomic status, and educational attainment make culturally derived communication signals inherently more complex reflecting the many nuances between the sender and recipient [24]. Both the aspiration to shared reality and the complexities inherent within medicine make the sole reliance on culturally general non-verbal signals an unlikely strategy for success. Because most non-verbal communication is unconscious [25], providers can default to signals they may not be aware of. This transition may or may not be in alignment with their intended message.

By contrast, medical school communication skills training focuses primarily on culturally generic signals like facing the patient or preferring sitting to standing when conducting the patient interview. Knowledge acquisition and performed behaviors are stressed [26]. But these techniques do not consistently foster a translation of learned interpersonal competencies to actual performance [27–30] and often do not lead to sustainable competences [28,29]. Such a focus even if successful presumes underlying empathy for the target group. Where patient and physician populations are very similar, this assumption is likely sound. However, different dynamics may operate when traditionally marginalized populations are under consideration. Here, a divergence between the theoretical capacity to practice empathetic behaviors and their daily performance may become apparent [29,31,32].

This present system incentivizes the performance of communication without interrogating or addressing underlying attitudes. None of the popular interventions offers a sustained modification of the likely biases from biographical experiences and learnings. We simply teach people to behave in a way they do not feel: a form of ruse. Some argue our efforts to teach empathy or communication skills explicitly encourage artificiality [33]. Such criticisms are not new [34,35]. Reductionist approaches may also impede the development of true communication expertise even when specific universal recommendations are retained [29]. Beyond bias against marginalized populations, there has also been little reckoning with the way the most common conceptions of physicians’ professional identity conflict with full engagement in the reflection and feedback needed to address interpersonal communication competence. The deep-seated defensiveness of students towards learning from their mistakes or their departures from professional ideals [36] frustrates the educational effort. Worse still, for a fraction of students that adopt recommendations despite all the above obstacles, dissonance develops between the negative beliefs they hold about certain patient populations and the more egalitarian views their formal instruction encourages. This deceptiveness has consequences as both a shortcoming for both the ways it limits the effectiveness of communication and the extent to which patients can appreciate it as dishonest.

Meaningful use of culturally specific signals is more than just awareness that such things exist. It involves recognition of the signal’s meaning within the broader context of the communication. Models of shared reality suggest that this goal is seldom achieved when the speaker is only responding to external pressures like a desire not to appear biased. Subtle but definite differences in emotional expression are evident across cultures. While some nonverbal behaviors appear universally desired in clinical environments, others show significant cultural variation [24,37]. Hence, cultural novices are unlikely to be cognizant of the range of cultural nuances and are likely to struggle to relate in cross-cultural contexts [19,38]. When cultural uncertainty is present, stereotypes are often substituted for knowledge and applied to stigmatized and marginalized patients [39]. Neither patients nor providers can take the most from an encounter when they miss these signals the other may have sent. Violating cultural norms of behavior in cross-cultural interactions impact patient expectations during doctor-patient interactions [40] and likely result in incomplete understandings, compromise empathetic accuracy [41] as well as the doctor-patient relationship [40,42]. The more familiar and accurate people become with specific cross-cultural emotional and nonverbal communication cues [43], the better physician-patient communications. Summarily, what the patient does not correctly receive, identify, or understand from the physician, diminishes the quality of the physician-patient interaction.

The damage to the physician-patient relationships incurred by the current approach depends to some extent on the patients’ ability to perceive dishonest communication. Incongruent nonverbal behavior or communication underlies most lie detection [44]. For example, there are detectable differences in facial muscle activation for nonverbal signaling with a sincere smile compared to a false one [45]. Whether through failure to appreciate or reflect culturally expected cues, patients likely appreciate physicians’ nonverbal incongruences with suspicion and may judge them as signs of dishonesty. Medical encounters, which can feature life-altering diagnoses or decision-making, represent high-stakes situations that make detection of insincerity and falsehoods more likely [46,47]. A study of informed consent found that despite identical content, African American patients rated the quality of communication as overall much poorer than did non-Hispanic Whites [48]. Such results demonstrate the possibility of divergence between verbal content and non-verbal communication and intent. Patients confronted with making a judgment about the sincerity of a smile make a risk assessment, often informed by their prior history of experiences with the physician and/or his culture. Their willingness to accept the risks they perceive from a physician is cumulative with each miscues tipping the scales further depending on the patient’s personality, outlook, and perceived ability to weather the consequences [49]. Regardless of underlying intention, perceived insincerity registers as a threat; these are more memorable, salient, and motivating to the patient than subsequent interpretations [50,51]. Deceptions, real or imagined, damage doctor-patient relationships, and compromise care [52].

Apart from limited didactic and haphazard clinical instruction typically with no follow-up, the remaining medical school opportunities are experiential. The underlying assumption is that culturally specific signals will be acquired through the practice of medicine by clinical exposure to diverse patients during training. These experiences are often filtered through a bias-condoning hidden curriculum, have limited duration or consistency. Further, we have not yet succeeded in controlling cultural microaggressions against our diverse learners [53–55], let alone making it a suitable environment for students to expand their ability to communicate with marginalized groups. The net-effect of diversity exposure on attitudes via contact is positive, but only when meaningful ties are established [20]. When this is difficult, as seen in segregated school systems or when individuals otherwise fail to build cross-cultural contacts it diminishes cross-cultural trust [56].^,^Unfortunately, many healthcare encounters more closely mirror these conditions for failure than those for success. Consequently, structured clinical activity does little for students’ understanding of stigmatized and marginalized people nor does it spur the development of the desired communication skills [57,58]. This array of factors against curricular efforts means none are likely to redress the communication deficits or the presence of outgroup bias. The gaps in the current approach are evident. The inter-personal communications instruction provided in medical school inadequately prepares students for the present and future cultural diversity they will encounter. Once upon a time, limited exposure was more serviceable because the broader society was more homogenous. That is no longer the case. We assume students have had diverse communicative experiences to draw on when the greater likelihood is that they do not. We trust communication skills will develop with clinical exposure without evidence that they can. We stress reductionist strategies, where concepts are eschewed in favor of specific behaviors like sitting during a patient interview. These approaches are short-sighted in that they homogenize, failing to account for the differences not only between but within cultures. The result is at best ineffective. What drives the development of humanism is authentic, unique, and participatory experiences [59]. The amount and quality of interactions needed to achieve that goal are dependent on intrinsic predispositions, biographical experiences and teachings, and cultural learnings of each individual. But an instructional program that fails to provide any at all misses the mark. Whether failing to incorporate these experiences or building capacity without evaluating underlying belief structures, the persistent lack of an over-arching strategy in medical school communications training renders it a bridge to nowhere.

Alternative tactics are imaginable. Interactions that are experiential yield greater gains than lectures or other passive learning formats [60]. Students acquire cultural fluency more slowly in small doses (e.g., medical encounters) than those who spend significant time immersed the different culture [10]. Contact between members of different groups in general, increases understanding and sensitivity while reducing intergroup bias [61,62] leading people to view each other as more similar reducing outgroup bias [63]. The greater and the more extensive the prior exposure to and interaction with another culture, the less unconscious bias [64], even if limited to a short engagement with exemplars of diverse social groups.

In other cases, existing approaches could be modified for more success. Medical language courses are offered in many schools, acknowledging the relationship of language to culture [65]. However, this has limited accessibility for those without previous understanding, and limited value for those who only memorize a few phrases. By contrast, the way an interpreter is utilized can increase both the empathy observed and the information exchanged in a medical encounter [66,67]. Perhaps the more fruitful path is instruction on how to work with interpreters to achieve these outcomes. Or consider the case of immersion experiences and dedicated clinics for marginalized populations. While global health experiences can be helpful, the medical problems of marginalized populations are local, not global. Although meaningful global cross-cultural experiences for Amsterdam medical students could be obtained in Germany or Turkey, the advantages of greater interactions with the local Turkish population make local sites favored. The potential differences in the environment faced by native versus immigrant populations and mean the experiences that animate behavior and communication in one locale will not be the same as that which motivates in other regions or countries. The common through line in the most effective exercises is either developing or capitalizing on experiential understanding and learning. Whether such activities are scalable for an entire medical school class is unproven. Most likely, the bulk of such experiences may have to be gained outside of medical school.

The idea that the rich tapestry of national cultures, each with its own cultural and contextual nuances can ever be reduced to a few rote universal gestures is simply misguided. Unlike the past when many of the patients were culturally like the practitioners who care for them, increasingly the opposite is true. Medical schools must act affirmatively to redesign communications training in a way that is cognizant of these weaknesses or use different selection factors for its students. We must move beyond miming techniques as communication training to a more fulsome contemplation of the underlying drivers of bias, ingroup/outgroup classification, and their resolution for effective communication.

The time is now to ensure that the physicians we produce can communicate effectively with all of society. Greater experiential exposure to the rich diversity of local cultures will likely need to take place before medical school. The distance to go before our graduates can provide quality care to local patients with whatever differences in backgrounds that are likely to exist is still formidable.
